# Comparison of Visually Guided Flight in Insects and Birds

**DOI:** 10.3389/fnins.2018.00157

**Published:** 2018-03-16

**Authors:** Douglas L. Altshuler, Mandyam V. Srinivasan

**Affiliations:** ^1^Department of Zoology, University of British Columbia, Vancouver, BC, Canada; ^2^Queensland Brain Institute, University of Queensland, St Lucia, QLD, Australia; ^3^School of Information Technology and Electrical Engineering, University of Queensland, St Lucia, QLD, Australia

**Keywords:** sensorimotor transformation, visuomotor control, flight speed, optic flow, image expansion

## Abstract

Over the last half century, work with flies, bees, and moths have revealed a number of visual guidance strategies for controlling different aspects of flight. Some algorithms, such as the use of pattern velocity in forward flight, are employed by all insects studied so far, and are used to control multiple flight tasks such as regulation of speed, measurement of distance, and positioning through narrow passages. Although much attention has been devoted to long-range navigation and homing in birds, until recently, very little was known about how birds control flight in a moment-to-moment fashion. A bird that flies rapidly through dense foliage to land on a branch—as birds often do—engages in a veritable three-dimensional slalom, in which it has to continually dodge branches and leaves, and find, and possibly even plan a collision-free path to the goal in real time. Each mode of flight from take-off to goal could potentially involve a different visual guidance algorithm. Here, we briefly review strategies for visual guidance of flight in insects, synthesize recent work from short-range visual guidance in birds, and offer a general comparison between the two groups of organisms.

## Overview of visual guidance algorithms for flight in insects

The principles of visual guidance for flight have been explored extensively in many insects. This research has revealed that insects rely heavily on optic flow—the pattern and speed of the motion of the image of the environment in their eyes that they experience during flight—to orchestrate a number of important behaviors (rev. Srinivasan, 2011a). A straight course and a stable attitude are maintained through the so-called “optomotor response,” which acts in such a way as to counteract rotations of the image of the environment in the eyes. The study of this optomotor behavior, pioneered by Hassenstein and Reichardt (1956) in the beetle *Chlorophanus*, has since been pursued in a wide range of insects, including the housefly *Musca*, the fruitfly *Drosophila*, (rev. Borst, 2009) and honeybees (rev. Srinivasan, 2011a). Honeybees (e.g., Srinivasan et al., 1996; Baird et al., 2005) and *Drosophila* (e.g., David, 1982; Fry et al., 2009) regulate the speed of their flight by monitoring and holding constant the optic flow generated by the surrounding environment. This ensures that the insect flies at a high speed in a safe, open environment (such as a field), and automatically slows down when it enters a cluttered environment such as a forest. Honeybees avoid collisions with objects by steering away from regions of the visual field that induce strong optic flow: rapid image motion signifies the presence of an object that is dangerously close (Kirchner and Srinivasan, 1989; Srinivasan et al., 1991; Srinivasan and Zhang, 1997). Honeybees (Kirchner and Srinivasan, 1989; Srinivasan et al., 1991) and bumblebees (Dyhr and Higgins, 2010) navigate safely through narrow gaps, avoiding collisions with the edges, by moving along a trajectory in which the two eyes experience the same magnitude of optic flow. *Drosophila* veer away from objects that generate rapidly expanding images that herald an imminent collision (e.g., Muijres et al., 2014). Honeybees orchestrate smooth landings by holding constant the magnitude of the optic flow that is generated in the vicinity of the landing target, as the target is approached (Srinivasan et al., 2000; Baird et al., 2013). This strategy ensures that the insect slows down progressively as the target is approached, reaching a speed that is close to zero at touch down. There is evidence that *Drosophila* control their deceleration while landing by measuring the rate of expansion of the image of the surface that is being approached (e.g., Van Breugel and Dickinson, 2012) and/or computing the time to contact from the image expansion (Wagner, 1982), and that the extension of the legs in preparation for the final touchdown (in houseflies and *Drosophila*) is trigged by the size and/or rate of expansion of the image of the surface that is being approached (e.g., Eckert, 1983; Borst and Bahde, 1986; Tammero and Dickinson, 2002; Van Breugel and Dickinson, 2012).

**Figure 1:**
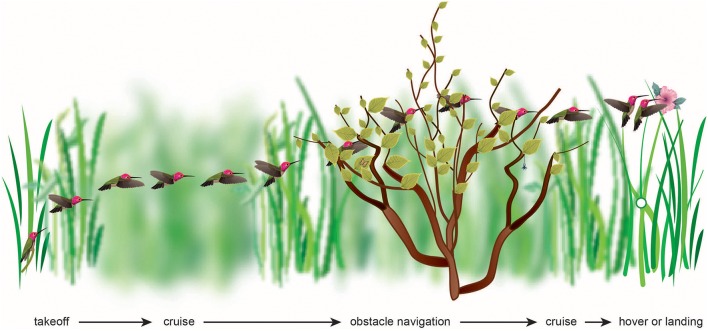
The flight of a bird through its natural environment requires that it make visually complex transitions, most of which have yet to be studied. The visual algorithms may include maintaining balanced optic flow or avoiding high optic flow, or maintaining constant velocity, acceleration, deceleration, height, rate of image expansion, or rate of change of time to collision.

Recent work indicates that birds, like insects, have strongly developed brain areas that are tuned for processing optic flow (revs. Frost, 2010; Wylie, 2013). Does this provide birds with equally adept visual flight control? What visual strategies do birds use while navigating through the environment, and how do these strategies compare with those known to be key for visual flight control in insects (Figure 1)? Until the past decade, relatively little research has been carried out to address these questions. Zebra finches hold their heads at a constant orientation -interspersed by brief saccadic rotations—while flying past an obstacle (Eckmeier et al., 2008). This suggests that optic flow could provide a reliable estimate of obstacle distance. How the information derived from optic flow is used to control the various phases of bird flight—such as takeoff, cruise, obstacle avoidance, and landing—remains to be uncovered, although some clues have begun to emerge, as will be described in this review.

Interestingly, the perception of movement appears to be “color-blind” in honeybees (Lehrer, 1987; Srinivasan, 2011a) as well as humans (Zeki, 1993), even though both creatures possess excellent trichromatic color vison. In the context of this article, it is of interest to enquire whether the movement-detecting pathways of birds—many of which possess tetrachromatic color vision—are also color-blind.

## Control of flight speed in budgerigars

**Figure 2:**
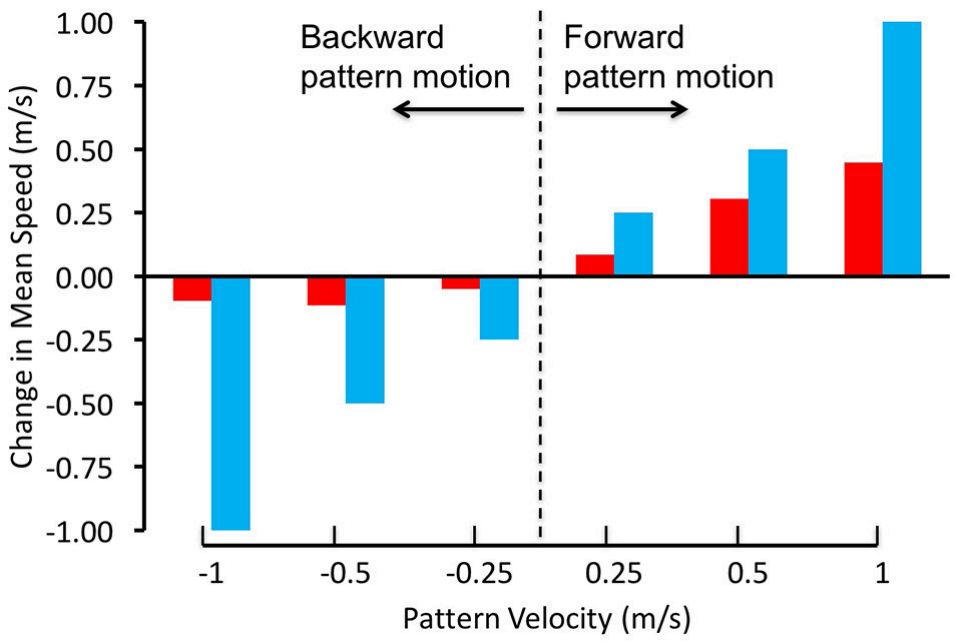
Regulation of flight speed of Budgerigars in a tunnel displaying moving gratings on the walls. The red bars denote the change in flight speed induced by the moving gratings in comparison with stationary gratings, where positive pattern velocities represent grating motion in the birds' flight direction and negative pattern velocities represent grating motion in the opposite direction. The blue bars represent the changes in flight speeds that would be expected if the Budgerigars matched the changes of their flight speed to the speeds of grating motion, i.e., if they held the rate of image motion constant in their eyes. Adapted with permission from Schiffner and Srinivasan (2015).

Schiffner and Srinivasan (2015, 2016) investigated the control and regulation of flight speed by flying Budgerigars in tunnels, and filming and reconstructing their flights in 3D using high-speed stereo video cameras. In their first study, the tunnel was of constant width, and displayed grating patterns that were projected on the side walls. The flight speeds of the birds were measured when the gratings on both walls were stationary, as well as in conditions when they were moved at a number of different speeds, both in the direction of flight and against it (Schiffner and Srinivasan, 2015). When the gratings were stationary, the birds flew through the tunnel at an average speed of about 6.3 m/s. When the gratings were moved in the direction of the birds' flight, at various speeds, the birds showed an increase in flight speed that was proportional to the increase in grating speed, but was not large enough to match it (Figure 2). When the gratings were moved in the opposite direction, the birds did not change their speed at all. Thus, Budgerigars do respond, to some extent, to changes in the motion of the image of the environment. However, unlike bees, these birds do not appear to rely solely on image motion to regulate the speed of their flight; other factors seem to play a role as well, as we shall discuss below.

**Figure 3:**
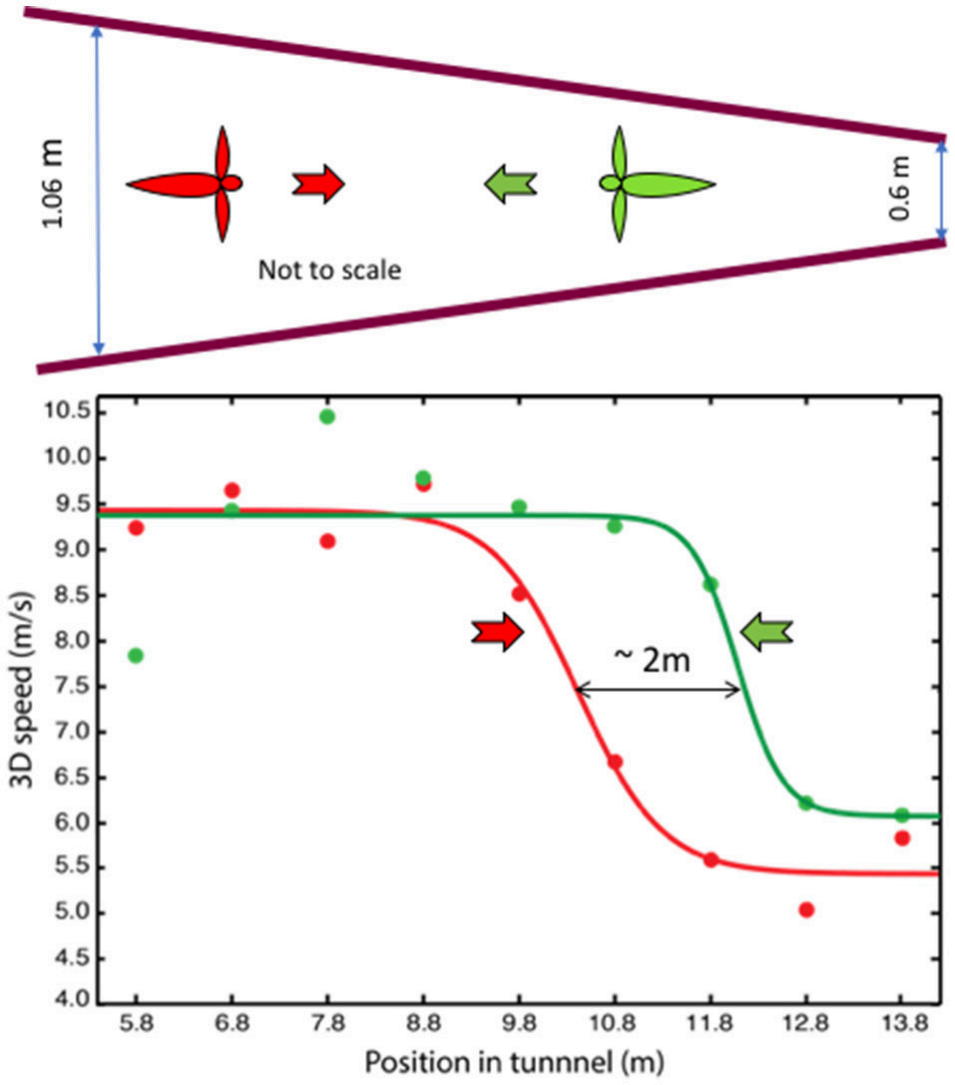
Profiles of flight speed of Budgerigars in a tapered tunnel, of height 2.4 m, during flight in the narrowing direction (red) and in the widening direction (green). Adapted with permission from Schiffner and Srinivasan (2016), which provides further information and statistical analyses of the results.

In a second study (Schiffner and Srinivasan, 2016), the tunnel carried stationary patterns on the walls, but the width of the tunnel varied gradually from one end to the other. Birds flying in this tunnel displayed only two, distinct flight speeds: a high speed of ~10 m/s in the wide section, and a constant low speed of ~5 m/s in the narrow section (Figure 3). Thus, like bees, Budgerigars fly faster in open environments and slower in narrow environments. However, while bees continuously adjust their speed during flight in a tapered tunnel—so as to hold the optic flow constant—Budgerigars seem to use primarily two distinct speeds.

These findings do not necessarily imply that optic flow is the visual cue that is used by the Budgerigars to gauge the width of the tunnel. Further investigation is needed to explore other possibilities. Given that the eyes of these birds possess laterally oriented fields of view with relatively little binocular overlap, stereo-based ranging is unlikely, and it is difficult to conceive of other plausible visual cues that could be used to assess tunnel width.

Why do Budgerigars behave differently from bees? We propose that, for Budgerigars (and possibly for other birds as well), energy requirements may play an important role in the choice of flight speed. The high speed measured in the tapered tunnel closely approximates the preferred (energy-optimum) cruising speed of Budgerigars, as measured in wind tunnel studies (~9.75 m/s; Tucker, 1968). The low speed, on the other hand, appears to be a speed that these birds adopt when they fly in relatively dark or cluttered environments. While it is not yet clear whether the low speed represents another local minimum of energy consumption (possibly a flight mode that involves flap-bounding), we find that our Budgerigars consistently fly at a low speed of ~5 m/s when they fly in environments that could be perceived by them to be unsafe. We therefore propose that Budgerigars use two flight speeds—a “cruising” speed of ~10 m/s in open environments, and a safe “maneuvering” speed of ~5 m/s during flight in dark or cluttered environments, switching between the two in response to changes that they encounter in the environment. Our results also reveal that when the birds fly through the tapered tunnel in the narrowing direction they switch to the low speed further into the tunnel, compared to the location at which they switch to the high speed during flight in the widening direction (Figure 3). The separation of ~2 m between these points suggests that the switching behavior is “anticipatory”: the birds are setting their speed by gauging the environment that is approximately 1 m ahead of their current position; that is, by sensing the magnitude of the optic flow in the regions of the visual field that are located about 20° lateral to the flight direction (Schiffner and Srinivasan, 2016).

## Control of flight through narrow passages

### Experiments using stationary gratings with budgerigars

Honeybees (Srinivasan et al., 1996) and bumblebees (Dyhr and Higgins, 2010) navigate safely through narrow corridors by flying close to the corridor's midline. Experiments have demonstrated that this is achieved by balancing the rates of image motion that are generated by the two walls during the flight through the corridor. An imbalance in these rates causes the insect to veer away from the wall that is generating the stronger optic flow. This “centering” response has been investigated experimentally in flying honeybees (Srinivasan et al., 1996), bumblebees (Dyhr and Higgins, 2010), and subsequently even in walking humans (Duchon and Warren, 2002) by moving the visual pattern on one wall or the other at various speeds, in the flight direction or against it. These manipulations reveal that safe steering through narrow corridors is achieved by balancing the flow signals (the image pattern velocities) that are experienced by the two eyes.

Another experimental approach to investigating the centering response has been to use stationary striped patterns on both walls. When both walls are decorated with vertically oriented stripes, the bees fly close to the midline of the tunnel. However, when one of the walls carries horizontal stripes, bees (Srinivasan et al., 1991) and bumblebees (Dyhr and Higgins, 2010) fly closer to that wall. This is consistent with the hypothesis that centering is achieved by balancing the magnitudes of optic flow generated by the two walls - the vertical stripes generate stronger optic flow, causing the subject to move closer to the horizontal stripes that generate weak or no flow because the direction of flight is parallel to the horizontal stripes.

Budgerigars behave in exactly the same way when they are subjected to a similar investigation with stationary stripes (Bhagavatula et al., 2011), suggesting that they, too, use a centering strategy that is based on balancing optic flow. However, the responses of these birds during flight in tunnels that present asymmetrically moving visual patterns is yet to be examined.

### Experiments using stationary and moving patterns with hummingbirds

One of the major advantages of behavioral studies with honeybees is that it is possible to study many individuals during natural flight, which allows for multiple tests over a range of experimental manipulations (Srinivasan et al., 1991, 1996). It can be more difficult to motivate birds to fly repeatedly in an experimental setting, but hummingbirds are one notable exception. These birds will fly often between a perch and an artificial feeder in an enclosed setting (e.g., Tiebout, 1991). Dakin et al. (2016) took advantage of this behavior to measure hummingbird flight trajectories in response to a variety of visual stimuli, including many of those previously tested with honeybees (Srinivasan et al., 1991, 1996).

Do hummingbirds also navigate through narrow corridors by balancing the magnitudes of optic flow generated by the two walls? To answer this question, Dakin et al. (2016) recorded hummingbird flight paths with moving patterns on the walls of a tunnel. The first experiment tested the response to vertical gratings with the pattern on one wall stationary, and the pattern on the other wall moving either toward or away from the feeder. Hummingbirds flew down the midline of the tunnel, indicating that they, unlike honeybees, did not control their lateral trajectory with respect to perceived pattern velocity. To confirm that this result was not due to the grating stimulus inhibiting a response, a second experiment tested the response to dot fields with the patterns on the two sides of the tunnel moving in opposite directions (i.e., one toward the feeder, one away). Again, hummingbirds did not adjust their lateral position in the tunnel relative to pattern velocity stimuli. To verify that the lack of responses in these two experiments was not due to a problem with the stimulus, a third pattern velocity manipulation tested the response to horizontal gratings moving symmetrically up or down. In this case, the birds did adjust their elevation: when horizontal gratings moved upwards, the birds flew at a higher elevation in the tunnel. This result indicates that at least one aspect of forward flight control is influenced by perceived pattern velocity.

Because hummingbirds did not adjust lateral position relative to manipulated pattern velocity, it is possible that hummingbirds and Budgerigars used different visual guidance strategies to navigate through a narrow passage. To evaluate this hypothesis, Dakin et al. (2016) tested hummingbirds with static gratings in the tunnel, vertical gratings on one side and horizontal on the other. The repeated tunnel passage behavior of hummingbirds allowed for a wide range of spatial frequencies to be tested (grating period size range 0.58–18.4 cm). The experiment revealed that hummingbirds flew on the side of the tunnel that was closer to horizontal gratings and away from the vertical gratings, consistent with a strategy of balancing optic flow, but only for an intermediate range of spatial frequencies of the paired gratings (period sizes 1.15 and 2.3 cm). When tested with very small (0.58 cm) or medium to large spatial period sizes (4.6–18.4 cm), the hummingbirds flew down the midline of the tunnel and thus, did not adjust their lateral position based on pattern velocity. The lack of response to very fine spatial frequencies (< ~5 cycles/°, Fellows, 2015) could be due to visual fusion (see Dakin et al., 2016, Figure 3) at the velocities at which the birds flew in the tunnel (2.0 m/s [95% CI 1.8, 2.2]). The lack of response to larger spatial frequencies could not be affected by visual fusion, and instead indicates that a different visual guidance strategy was used.

**Figure 4:**
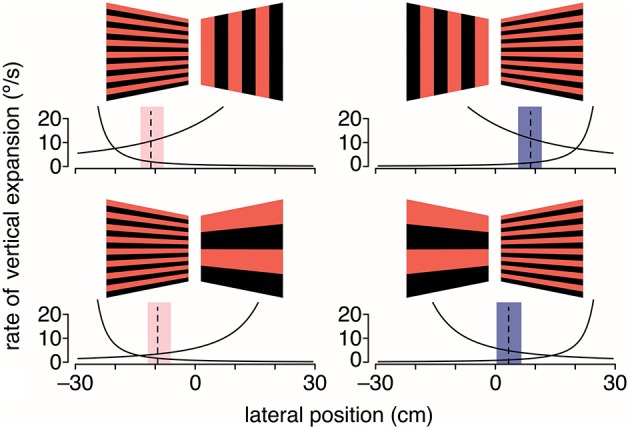
Hummingbirds appear to use expansion cues for lateral course control. Black and red gratings depict the stationary visual patterns displayed on the left and right walls of the tunnel. Dashed lines are the average lateral positions, and shaded regions are the average extremes for birds at the halfway point through the tunnel. Black lines indicate the rate of vertical expansion for a bird moving laterally at 0.1 m/s, which is the typical maximum lateral flight speed. Adapted with permission from Dakin et al. (2016).

Given that gratings with medium to large spatial frequencies did not lead to the expected shift in flight trajectory, it is possible that either large vertical gratings are less repulsive or large horizontal gratings are less attractive. To evaluate this question, hummingbirds were again tested with stationary gratings but with grating pairs that differed in spatial frequency (Figure 4). When large (18.4-cm period size) vertical gratings were paired with intermediate (2.3-cm period size) horizontal gratings, hummingbirds flew closer to the horizontal gratings. In contrast, when the stimulus consisted of large horizontal and intermediate vertical gratings, hummingbirds flew down the center of the tunnel (Dakin et al., 2016). In the final test, the stimulus consisted of intermediate horizontal gratings on one side and large horizontal gratings on the other. In response, hummingbirds flew much closer to the intermediate horizontal gratings. Collectively, these results are consistent with the hypothesis that hummingbirds are using a strategy of balancing the rate of visual expansion, rather than balancing pattern velocity, to control their lateral flight trajectory (Figure 4). Confirmation of this hypothesis will require additional experiments that manipulate the perceived rate of image expansion.

Are the different results between tunnel studies with Budgerigars (Bhagavatula et al., 2011) and hummingbirds (Dakin et al., 2016) due to species-specific differences or due to different experimental tests? Answering this question will obviously require a larger set of experimental treatments to be performed with both species. However, a key idea is there was a set of spatial frequencies for stationary gratings for which the Budgerigar and hummingbird results were similar. This suggests that both species could be using the strategy of balancing rate of vertical expansion, and that when the vertical expansion cue differences between left and right sides are substantial enough, the response appears consistent with the pattern velocity strategy. Another possibility is that Budgerigars, which are unable to sustain hover, rely more on cues derived from translational image motion, rather than expansional image motion, to center their flight through narrow passages. Because the stimulus pairs tested so far with hummingbirds have not yet been tested with honeybees (Srinivasan et al., 1991, 1996), it would be intriguing to ask if insects are also using vertical expansion cues.

## Control of avian flight through cluttered environments and very narrow passages

During flight in dense forests, birds often need to fly through extremely narrow passages without hurting themselves. Guidance of flight through a two dimensional array of obstacles (vertical poles), akin to a forest of trees, has been investigated in the laboratory in pigeons (Lin et al., 2014). The birds had the option of taking several different routes of flying through the constellation of obstacles. This study revealed that the birds tended to choose routes in which the successive gaps that were encountered were (a) as wide as possible, and (b) consistent with the desired flight direction. Similar results were obtained by Ros et al. (2017), in which pigeons were again trained to fly through a two-dimensional array of obstacles, but where the obstacles were oriented horizontally, rather than vertically. This required the birds to fly through the obstacle constellation by rapidly varying their flight height, rather than changing their flight direction. Here again, the birds tended to choose routes in which the gaps that were encountered were as wide as possible, and consistent with the desired flight direction.

**Figure 5:**
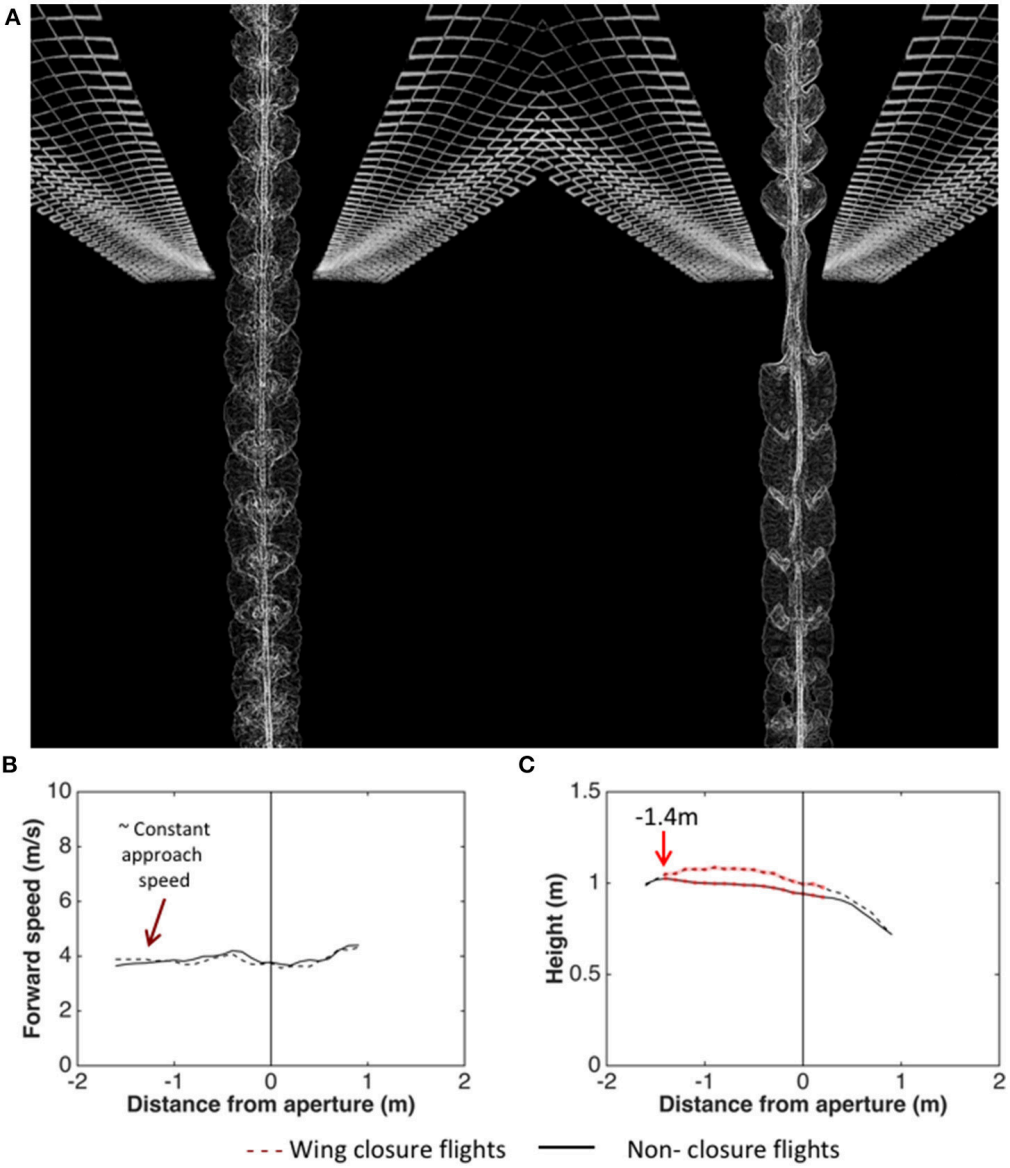
**(A)** Video-based visualization of Budgerigar flight through an aperture that is wider than the wingspan (left) and narrower than the wingspan (right). Image courtesy H. Vo and I. Schiffner. **(B,C)** Analysis of trajectories of Budgerigars approaching apertures of various widths, comparing flights through apertures that require wing closure (dashed curves) with flights through apertures that do not require wing closure (solid curves). Left: Mean profiles of flight speed; Right: Mean profiles of height. Adapted from Vo et al. (2016).

Schiffner et al. (2014) investigated the kinematics of flight through a single aperture by training Budgerigars to fly through a corridor which presented a single narrow, vertically oriented aperture (a slit), mid-flight. The birds' flights were video-filmed as they flew through the aperture for various aperture widths - ranging from several times the wingspan, down to values just marginally greater than the width of the thorax. The results revealed that, during the passage through the aperture, they close their wings if (and only if) the wingspan exceeds the aperture width. Figure 5A illustrates a comparison of a flight through a wide aperture, with a flight through an aperture that is narrower than the wingspan. Analysis of the wing-closing behavior of Budgerigars flying through apertures of various widths reveals that these birds are aware of their wingspan to a precision of ± 1 cm, which is about 6% of the average wingspan (Schiffner et al., 2014). Furthermore, analysis of the behavior of individual birds (with wingspans that vary over a range of 29–33 cm across the group of birds tested) reveals that wing closure is not triggered at a fixed aperture width. The critical aperture width varies from bird to bird, and depends upon its individual wingspan. Thus, each bird is precisely aware of its personal wingspan (Schiffner et al., 2014).

Wing closure, when it occurs, occurs well ahead of the aperture—at a modal distance of about 22 cm. Thus, the cues that are used to determine whether wing closure is necessary—and if so, to determine the distance at which it occurs—are most likely based on vision, and not on tactile or aerodynamic interactions with the aperture.

A later study (Williams and Biewener, 2015) investigated the same question in pigeons and found similar results, with the additional observation that these birds adopted one of two postures while passing through narrow gaps. When the gap was very narrow, they folded their wings back completely (like the Budgerigars). When the gap was wider (but still narrower than the wingspan), they adopted a “paused” posture in which the wings were held stationary at the top of the upstroke. Here again, the birds were clearly aware of the size of their wingspan in relation to the width of the gap.

Why do birds need such precise knowledge of their wingspan? We suggest that Budgerigars and pigeons interrupt the flapping of their wings only when it is absolutely necessary because such an interruption (a) reduces the lift force that is necessary to keep the bird aloft, and (b) seriously compromises the ability to control flight, effectively reducing the bird to a projectile. It would be interesting to ask whether this type of “body awareness” in birds is genetically pre-programmed, or learnt from experience and updated steadily as the bird grows to an adult—the latter perhaps being a more likely explanation. One approach to answering this question would be to look for changes in wingspan awareness when the wingtips are trimmed or artificially extended. One has to bear in mind, however, that such manipulations could affect the weight and aerodynamics of the wings in artificial ways, complicating the interpretation of the results.

Analysis of the trajectories of the birds as they approach the apertures reveals that the birds which fly through the aperture without closing their wings (because the aperture is wider than the wingspan) maintain a constant height throughout the approach (Figure 5B; Vo et al., 2016). On the other hand, the birds that close their wings (because the aperture is narrower than the wingspan) increase their height at a mean distance of about 140 cm from the aperture (Figure 5C; Vo et al., 2016). This increase of height occurs well ahead of the point at which the wing closure actually occurs (22 cm from the aperture; Schiffner et al., 2014). We suggest that this height increase is a manoeuver to compensate for the subsequent loss of height that occurs due to the temporary closure of the wings. If this is true, it would imply that Budgerigars pre-plan flights through narrow gaps in a sophisticated way.

Analysis of the approach trajectories also reveals that the speed at which the birds approach the aperture is independent of the width of the aperture, and is independent of whether or not the aperture requires wing closure (Vo et al., 2016). The birds approach all of the apertures at a speed of approximately 4 m/s (Figure 5B). Interestingly, this speed is close to the “maneuvering” speed that the birds display in the narrower part of the tapered tunnel (see Figure 3). This reinforces the notion that Budgerigars fly at a stable, constant speed of 4–5 m/s during flight in cluttered environments. Exactly how cluttered or “dangerous” the environment has to be to trigger a switch to the lower speed, and exactly what properties of the environment are used in making this decision, remains to be investigated.

Why do Budgerigars adopt a constant flight speed in dense environments? One potential advantage of this strategy is that moving at a constant, known speed allows the range to various obstacles in the environment to be calibrated directly in terms of the optic flow that is experienced by the visual system. This appears to be different from the behavior of most flying insects, including bees, which progressively reduce their flight speed as the density of the environment increases (Srinivasan et al., 1996). Therefore, unlike Budgerigars, flying insects face a “chicken and egg” problem: The distance to an object cannot be estimated without knowledge about the flight speed, and vice versa. Nevertheless, insects have evolved ingenious solutions to overcome this paradox in the context of many visually guided behaviors such as cruising flight, obstacle avoidance and landing (Srinivasan, 2011b).

The findings with the Budgerigars beg the question of how these birds regulate their speed at each of the two speeds at which they prefer to fly. The higher speed, which is in the vicinity of 10 m/s, may be set by the energy consumption curve, which exhibits a minimum at this speed (Tucker, 1968). The mode of regulation at the lower speed remains to be explored. Two possibilities are (a) generating a constant, calibrated thrust; and (b) sensing the airspeed (possibly via the feathers) and using this information to control thrust in a feedback loop.

## Mid-air collision avoidance by budgerigars

During flight, birds need to avoid collisions with stationary obstacles as well as moving objects, such as other birds. Schiffner et al. (2016) investigated mid-air collision avoidance in Budgerigars by launching two birds from the opposite ends of a narrow tunnel and video-filming their flights as they approached and flew past each other. The results revealed that Budgerigars avoid imminent head-on collisions by adopting a simple, consistent rule: each bird veers to its right. Evidently this strategy for collision avoidance—which is also used by airplane pilots—has been evolved by these birds millions of years ago. It remains to be explored whether this “right hand rule” applies to other bird species as well.

## Deceleration and hovering

### Target approach and docking

Observations of birds approaching targets such as gannets diving to capture fish in water (Lee and Reddish, 1981), hummingbirds docking at flowers (Lee et al., 1991) and pigeons landing (Lee et al., 1993) suggest that they control their approach by calculating time to contact. These observations are consistent with a broad strategy of visually guiding target approach via time to contact, which is described in detail elsewhere (Lee, 1976, 2009). However, we note that neurons with response properties required to calculate time to contact and its rate of change have been identified and studied in the nucleus rotundus of pigeons (Sun and Frost, 1998).

### Holding station

Most birds do not have the hovering capabilities of insects, although many species can transiently hover to search for or consume food. The notable exception is the hummingbird (Family Trochilidae), which can sustain hovering during long periods for nectar consumption, feeding on small arthropods, and surveillance. Although the transient hovering of other species is challenging to study in the laboratory, hummingbirds will readily hover in controlled settings allowing for the investigation of their physiology (Lasiewski, 1963), biomechanics (Stolpe and Zimmer, 1939; Wells, 1993; Chai and Dudley, 1995), and, most recently, visual guidance (Goller and Altshuler, 2014; Ros and Biewener, 2016; Goller et al., 2017).

When attempting to hold station, all visual animals studied to date exhibit motion drift in response to experimentally produced global optic flow, i.e., an optomotor response. Such self-motion or attempted self-motion (for restrained animals) represents an attempt to minimize visual motion on image forming eyes. This strategy is also used by flying hummingbirds attempting to hover in virtual reality chambers with projected optic flow (Goller and Altshuler, 2014; Ros and Biewener, 2016). However, there is an aspect of this behavior that may be unique to hummingbirds, at least compared to other tetrapod species studied to date. Whereas many other tetrapods, including humans (van den Berg and Collewijn, 1988), cats (Markner and Hoffmann, 1985), rabbits (Erickson and Barmack, 1980), rats (Hess et al., 1985), chicks (Wallman and Velez, 1985), pigeons (Gioanni, 1988), and turtles (Hertzler and Hayes, 1969), have enhanced gain in their optomotor responses to temporal-to-nasal (back-to-front) visual motion, hummingbirds respond more or less equally to motion in all six major directions: left-to-right, right-to-left, up-to-down, down-to-up, back-to front, and front-to-back (Goller and Altshuler, 2014).

The difference in optomotor response between hummingbirds and other tetrapods may be explained by the response properties of neurons in the nucleus lentiformis mesencephali (LM). The LM and homologous nucleus of the optic tract (NOT) of mammals contains neurons that respond to wide-field visual motion. The global motion neurons are activated most strongly in a preferred direction and are suppressed in the opposite (null) direction. Recordings from diverse tetrapod species, including monkeys (Mustari and Fuchs, 1990), rabbits (Collewijn, 1975), wallabies (Ibbotson et al., 1994), cats (Hoffmann and Schoppmann, 1981), chicks (McKenna and Wallman, 1981, 1985), pigeons (Winterson and Brauth, 1985; Wylie and Crowder, 2000), turtles (Fan et al., 1995), salamanders (Manteuffel, 1984), and frogs (Katte and Hoffmann, 1980; Li et al., 1996), demonstrate that the majority of LM and NOT neurons prefer temporal-to-nasal (or back-to-front) motion. The hummingbirds LM differs in two ways. First, this nucleus is hypertrophied relative to all birds species (Iwaniuk and Wylie, 2007). Second, although individual hummingbird LM neurons have direction preferences, as in other tetrapods, as a population there are neurons responding to all directions with no overall direction bias (Gaede et al., 2017). Therefore, both the visual guidance of hummingbird hovering flight and the electrophysiological response properties of hummingbird global visual motion neurons indicate enhanced specialization for detecting and responding to visual motion in multiple directions.

## Color blindness of movement perception

Classically, movement-induced responses in flying insects have been studied by tethering the insect in the middle of a drum, decorated with vertically oriented black-and-white stripes, and measuring the insect's yaw torque when the drum rotates alternately clockwise and counterclockwise. These studies, pioneered by Reichardt (1969), have since been carried out in a wide range of insects, including the housefly *Musca*, the fruitfly *Drosophila*, and honeybees (Apis), references for which are given in Srinivasan (2011a). Under these conditions the insect attempts to follow the rotation of the drum, exerting a clockwise torque when the drum rotates clockwise, and vice versa. In free flight, these turning (“optomotor”) responses serve to compensate for unintended (disturbance induced) deviations from the intended flight direction (Reichardt, 1969). Interestingly, honeybees in this experimental paradigm display strong optomotor responses when the alternating stripes are black and white, but no response at all when the colors and intensities of the alternating stripes are such that they excite the honeybee's green receptor equally strongly (Kaiser, 1975), thus providing no detectable contrast to the green receptors. This finding and a number of other observations (Lehrer, 1987; Srinivasan, 2011a) suggest that the movement detecting pathways in the bee's visual system are driven exclusively by the green receptor channel and are therefore “color blind”—although the bee's color perception pathway endows it with excellent trichromatic color vision, featuring signals from UV-, blue- and green-sensitive photoreceptors.

When bees are trained to land on a disc placed on a table to feed on a drop of sugar water positioned at the center, they touch down at the edge of the disc and walk to the reward, rather than land directly at the reward (Lehrer et al., 1990). Evidently, bees aim for the boundary at the rim of the disc, and use this high-contrast visual feature to guide their landing. Bees land consistently at the rim of the disc when the disc is black and the background (table) is white. However, if the colors of the disc and the background are such that they provide no contrast to the green receptors, the bees no longer land selectively at the rim of the disc (Lehrer et al., 1990). This demonstrates that the visual cue used to guide the landing at the target is sensed by a movement-detecting pathway that is again color blind.

In the Budgerigar, the chromatic properties of movement detection were investigated by using a similar landing paradigm in which the birds were trained to land and collect food placed at the center of a blue disc (Bhagavatula et al., 2009). Here again, the birds, like the bees, generally tended to land at the rim of the disc. However, when the color of the disc was held constant and the background was varied from black to white through various shades of gray, the birds lost their preference for the rim when the background was a particular shade of gray that stimulated the bird's red photoreceptors exactly as strongly as did the blue disc. This finding suggests that, in Budgerigars, the movement-detecting pathway that guides landings is again color blind, and is in this case driven by the red photoreceptors (Bhagavatula et al., 2009). Further work is required to investigate the chromatic properties of other movement-sensitive behaviors in birds.

Interestingly, movement perception is color blind in humans as well: it is driven by the luminance pathway, which sums signals from the red and green cones (Zeki, 1993). Why is movement detection color blind across several animal species, as the evidence so far seems to suggest? One possible explanation is that the capacity to detect and respond to movement—which is critical to many aspects of behavior—is a fundamental building block that visual systems, initially working with a single spectral class of photoreceptor, evolved as part of their basic “Bauplan,” before incorporating additional capabilities such as color vision (Srinivasan, 2011a).

## Conclusions

There has been considerable work on the visual guidance strategies of flying insects, particularly with honeybees. Recent work with flying birds points to some similarities in the strategies used for flight control, although with substantial modification. For example, movement perception appears to be color-blind for both groups, but with different cone pigments in birds compared to insects. However, work so far indicates more differences than similarities in flight strategy, with key examples being the centering response and the control of flight speed. In both of these cases, the avian strategies seem to be less stereotyped than in insects. Centering and velocity control during forward flight in insects relies primarily on pattern velocity cues, whereas birds adjust position and speed during forward flight to a combination of pattern velocity and the rate of vertical image expansion.

Could the differences in visual guidance strategies between flying birds and insects be due simply to variation in experimental approaches? For example, in many of the insect studies, the optic flow was manipulated in the ventral as well as the lateral regions of the visual field, whereas in the case of birds, this manipulation has so far been restricted to the lateral fields. Another, broader reason may have to do with the fact that birds, given their higher visual acuity and more developed brains, possess better “scene awareness” than do insects, making it experimentally more challenging to change their behavior by manipulating the visual stimuli that they experience. It would be highly informative to repeat a larger set of the same experimental treatments for both groups.

A major driver of the recent efforts to study visual guidance in birds was the availability of tracking systems and large field stimulus presentation. At present, such systems are not well automated but it is likely that with greater availability and usability, we will know much more about avian visual guidance in the coming years. A potentially fruitful avenue of investigation would be to combine the behavioral studies with electrophysiological investigations of the neural substrates. Behavior and electrophysiology have long been integrated in research with insects, and when combined with molecular genetics, this has led to major progress over the last decade in understanding visual circuits for flight control (Rister et al., 2007; Joesch et al., 2010; Maisak et al., 2013; Behnia et al., 2014). Because molecular approaches now available in non-genetic model organisms are starting to be used with birds (Roberts et al., 2012, 2017), we are hopeful that a deep understanding of how the avian brain transforms visual information into motor output is within view.

## Author contributions

DA and MS wrote and edited the manuscript.

### Conflict of interest statement

The authors declare that the research was conducted in the absence of any commercial or financial relationships that could be construed as a potential conflict of interest.
